# Prevalence of intestinal parasites and comparison of detection techniques for soil-transmitted helminths among newly arrived expatriate labors in Jeddah, Saudi Arabia

**DOI:** 10.7717/peerj.16820

**Published:** 2024-01-26

**Authors:** Mohammad F. Al-Refai, Majed H. Wakid

**Affiliations:** 1 Medical Microbiology, Department of Medical Laboratories, King Fahd Armed Forces Hospital, Jeddah, Saudi Arabia; 2 Faculty of Applied Medical Sciences, Medical Laboratory Sciences Department, King Abdulaziz University, Jeddah, Saudi Arabia; 3 Special Infectious Agents Unit, King Fahd Medical Research Center, Jeddah, Saudi Arabia

**Keywords:** Intestinal parasites, Soil transmitted helminths, Detection techniques, Microscopic examination, Chromatographic immunoassay, Real-time PCR, Expatriate labors, Jeddah, Saudi Arabia

## Abstract

**Background:**

Diversity in clinical signs and symptoms are associated with soil transmitted diseases (STD), which are spread to humans by intestinal worms and transmitted in a variety of ways. There is a need for the present study, which aimed to investigate the prevalence of intestinal parasites and to compare between the common detection techniques for soil-transmitted helminths (STHs) among newly arrived expatriate labors in Jeddah, Saudi Arabia.

**Methods:**

A total of 188 stool samples were analyzed by macroscopic examination, and microscopic examination using direct iodine smear and the formal ether sedimentation technique. Trichrome and modified Kinyoun’s stains were used to confirm the morphology of any detected protozoa stages and oocyst of *Cryptosporidium*, respectively. A chromatographic immunoassay kit was used for *Entamoeba histolytica*, *Giardia lamblia* and *Cryptosporidium*. In addition, real-time PCR was employed only to identify various STHs.

**Results:**

Out of 188, several types of parasites were detected in 35 samples (18.62%), of which some with multiple infections. Nine samples (4.79%) were positive for *Entamoeba coli*, seven samples (3.72%) for *Trichuris trichiura*, six samples (3.19%) for *Necator americanus*, four samples (2.13%) for *Strongyloides stercoralis*, four samples (2.13%) for *Ascaris lumbricoides*, four samples (2.13%) for *E. histolytica*, three samples (1.60%) for *Blastocystis hominis* and two samples (1.06%) for *Ancylostoma duodenale*. In comparison between laboratory techniques for STHs, real-time PCR was able to detect the DNA of 19 samples (10.1%) followed by Ritchie sedimentation technique (18, 9.6%), and direct smear (7, 3.7%) (*p* > 0.05).

**Conclusion:**

The high rate of newly arrived foreign workers infected with intestinal parasites could lead to a risk to society. Continuous and regular surveys are needed to deal with the occurrence of intestinal parasitic infections including STHs. To improve the identification of these infections, we recommend a supporting infrastructure for the application of concentration methods and molecular assays.

## Introduction

Infectious intestinal parasites, which include helminths and protozoans, may cause several serious health problems such as malnutrition, anemia, and cancer, especially in impoverished and tropical countries (World Health Organization, 2002; Torgerson et al., 2015). More serious complications are seen in immunocompromised patients (Bahwaireth & Wakid, 2022; Omrani et al., 2015). Several serious waterborne outbreaks in the world were caused by parasites (Efstratiou, Ongerth & Karanis, 2017).

According to the World Health Organization (2020) (WHO), in 2020 roughly 25% of the global population was infected with soil-transmitted helminths (STHs), while over 3 billion revealed no symptoms. These STHs belong to the Phylum Nematoda (roundworms), and include *Ascaris lumbricoides*, hookworm (*Ancylostoma duodenale* and *Necator americanus*), *Strongyloides stercoralis* and *Trichuris trichiura*. Contaminated soil plays a vital role in STHs transmission and life cycles (Wakid, 2020b).

*Blastocystis hominis, Entamoeba histolytica Giardia lamblia*, and *Cryptosporidium* are the common intestinal protozoan parasites. According to the WHO, in the developing world, *G. lamblia* is the most pervasive parasite that causes diarrhea, while the invasive amoebic infection affects approximately 50 million people every year, resulting in 40–100 thousand deaths (World Health Organization, 1997). The majority of intestinal protozoa spread through fecal contamination caused by poor sanitation and contaminated water or food (World Health Organization, 2006; CDC, 2019; Cama & Mathison, 2015).

Infection with non-pathogenic intestinal protozoa such as *E. coli, Endolimax nana* and *Iodamoeba buetschlii* should be reported, which indicates a lack of hygiene, as their route of transmission is similar to the pathogenic protozoa.

The increased risk of getting intestinal parasites is due to a variety of socioeconomic, environmental, and hygienic variables. Rapid socioeconomic progress and long-term economic stability, on the other hand, have resulted in a massive migration of expatriate labor, primarily from less wealthy and developed nations (Baker et al., 2022). Within a population, parasitic infection patterns vary and are connected to origin nations (Blair & Sharif, 2012; Arfaa, 1981; Salas, Heifetz & Barrett-Connor, 1990; Varkey et al., 2007; Abu-Madi, Behnke & Ismail, 2008), host gender (Stephenson, Latham & Ottesen, 2000), and yearly changes in parasite transmission rates within a population pool (Varkey et al., 2007; Wang, 1998; Al-Shammari et al., 2001).

The Kingdom of Saudi Arabia (KSA) is a fast-expanding country with a diverse population from a wide range of educational backgrounds, religious views, eating and leisure habits and behaviors, and cultural customs. In 2021, the KSA’s population was around 34 million, with a growth rate of 1.5% (General Authority for Statististics, 2021; The World Bank, 2021). The majority of expatriates to the KSA come from underdeveloped nations in Africa and South Asia (UNICEF, 2014; United Nation, 2021), where parasitic illnesses are common (Cross & Basaca-Sevilla, 1981; Ward, 2009; Abubakar, Tillmann & Banerjee, 2015). Over 80% of the workforce in the KSA is made up of expatriates from Asian countries (Expatriate Population, 2018).

The diagnosis of intestinal parasites including STHs is mainly based on the microscopic examination of stool samples by using different techniques. Recently, the molecular diagnosis by real-time PCR has provided a more sensitive and specific laboratory method (World Health Organization, 2019; Garcia et al., 2017).

Using improved and cost-effective diagnostic tools as a routine diagnostic strategy helps to minimize the under-diagnosis and underreporting of intestinal parasites including STHs infections. This will be supportive for more feasible preventive and control measures. Therefore, the aim of this study was to investigate the prevalence of intestinal parasites, and to compare the microscopic (direct smears, sedimentation concentration) and molecular (real-time PCR) techniques for the detection of STHs among newly arrived expatriate labors in Jeddah, Saudi Arabia.

## Materials and Methods

This study was conducted according to the guidelines of the Declaration of Helsinki and approved by The Ethics and Research Committee of the Faculty of Applied Medical Sciences, King Abdulaziz University, (FAMS-EC2018-012), and written informed consent was obtained from each participant.

### Samples collection and study group

This cross-sectional study included newly arrived expatriate labors in Jeddah regardless of gender and age. Stool specimens were collected from a laboratory of expatriate workers polyclinic between January and March 2018. Each participant was handled a container and guidelines to collect the stool specimen. A total of 210 specimens were collected, but after checking the quantity of the stool, 22 specimens were excluded from the study due to insufficient amounts. According to that, a total of 188 specimens were used for this study.

The collected samples were refrigerated, and then investigated through gross examination, light microscopy and real-time PCR on a daily basis.

The laboratory investigation was conducted at the Diagnostic Parasitology Laboratory in the Special Infectious Agents, King Fahd Medical Research Centre at King Abdulaziz University in Jeddah, Saudi Arabia.

### Macroscopic examination

Stool specimens were examined grossly to determine the consistency (hard, formed, loose or watery), color and presence of blood, mucus, and gross parasitic stages such as segments of tapeworms or whole adult worms.

### Microscopic examination by direct smears

For a direct smear, about 1–2 mg of stool was mixed by wooden stick with 1–2 drops of Lugol’s’ iodine on a clean glass slide, then mixed and covered by a 22 mm × 22 mm coverslip. The slide was examined under 10× and 40× objective lenses of the light microscope.

### Microscopic examination by sedimentation technique

Ritchie formal-ether sedimentation technique was performed by emulsifying 2 gm of stool in 10 ml of 10% formal saline. The suspension filtered *via* two layers of gauze fitted to a funnel into a 15 ml centrifuge tube, then centrifuged for 5 min at 2,000 rpm. The supernatant was discarded, and the sediment was re-suspended with 10 ml of formal saline (10%). After that, 3 ml of diethyl ether was added, and the tube was shaken vigorously for 15 s, and then centrifuged for 5 min at 2,000 rpm. Four layers were observed, the diagnostic stages were precipitated at the bottom of the tube, and the debris formed a layer separated between diethyl ether and formal saline. A wooden stick was used to detach the debris layer, and the tube rapidly inverted to decant the unwanted layers. Finally, the sediment was mixed with two drops of iodine and a drop was placed on a microscope slide and covered with 22 mm × 22 mm cover glass, then examined under 10× and 40× objective lenses.

### Microscopic examination by trichrome permanent staining

To confirm the morphology of the protozoan parasites, PVA-preserved stool samples were stained with Para-Pak® trichrome kit, according to the manufacturer’s instructions, and then examined microscopically by oil immersion objective lens as described previously (Aldahhasi, Toulah & Wakid, 2020).

### Microscopic examination by modified kinyoun’s staining

This stain was used to detect *Cryptosporidium*. A stool smear was prepared from each sample and allowed to air dry, then fixed with methanol. After that, the smear was stained for 5 min with carbol-fuchsin. Each smear was then washed with tap water, decolorized for 2 min in acid alcohol, washed with tap water, and then counter-stained for 5 min with methylene blue. Finally, the stained smear was washed with tap water, air dried, and examined under the light microscope (Garcia et al., 2017).

### Rapid chromatographic immunoassay test

A previously described, ImmunoCard STAT! CGE was performed according to manufacturer’s instructions to detect *Giardia lamblia, Cryptosporidium*, and *Entamoeba histolytica* antigens by the monoclonal antibodies (Alharbi et al., 2020).

### Molecular diagnosis (real-time PCR)

The DNA was extracted from stool samples (stored at −20 °C) by using the QIAamp Fast DNA Stool Mini Kit as described by the manufacturer’s instructions (QIAamp, 2020), then kept at −20 °C until use.

The extracted DNA was amplified and detected for STHs specifically. Target genes included the internal transcribed spacer (ITS1) was used for *A. lumbricoides* and *T. trichiura* assays, and the ITS2 for *A. duodenale* and *N. americanus* assays (GenBank accession nos. AJ000895, FM991956, AJ001594, and AJ001599, respectively) (Taniuchi et al., 2011; Mejia et al., 2013). Furthermore, the 18S ribosomal RNA (rRNA) gene was used for *S. stercoralis* (GenBank accession no. AF279916) (Taniuchi et al., 2011). All the primer sequences (5′ to 3′) used in the present study are listed in (Table 1).

**Table 1:** Primers and probe for real-time PCR. All the oligonucleotide sequences (5′ to 3′) used in the present study.

Parasite name/primer		Sequences
*A. lumbricoides* ITS-1	F	GTAATAGCAGTCGGCGGTTTCTT
	R	GCCCAACATGCCACCTATTC
	P	TTGGCGGACAATTGCATGCGAT
*A. duodenale* ITS-2	F	GAATGACAGCAAACTCGTTGTTG
	R	ATACTAGCCACTGCCGAAACGT
	P	ATCGTTTACCGACTTTAG
*N. americanus* ITS-2	F	CTGTTTGTCGAACGGTACTTGC
	R	ATAACAGCGTGCACATGTTGC
	P	CTGTACTACGCATTGTATAC
*T. trichiura* ITS-1	F	TCCGAACGGCGGATCA
	R	CTCGAGTGTCACGTCGTCCTT
	P	TTGGCTCGTAGGTCGTT
*S. stercoralis* 18S rRNA	F	GAATTCCAAGTAAACGTAAGTCATTAGC
	R	TGCCTCTGGATATTGCTCAGTTC
	P	ACACACCGGCCGTCGCTGC

The master mix, working primers and probe solutions were prepared to the specifications previously described (Alqarni, Wakid & Gattan, 2022), and stored at −20 °C until use.

The reaction mix was dispensed in appropriate volume of 15 μl into each well of PCR reaction plate containing 96-well. Then 5 μl of the DNA was added to the individual wells containing the reaction mix. Negative control was used in in every run. The plate was then transferred into Applied Biosystems 7500 Fast real-time PCR system, to start the cycling program and perform data analysis. Real-time PCR conditions were set up according to QuantiTect® Probe PCR kit instructions as shown in (Table 2).

**Table 2:** The real-time PCR conditions set up according to QuantiTect® Probe PCR kit.

Step	Initial activation	Denaturation	Combined annealing/extension
Temp	95 °C	94 °C	60 °C
Time	15 min	15 s	1 min
Cycling	1 cycle	45 cycles	

### Statistical analysis

Categorical data was reported as frequency, cross-tabulation, and percentage (%). Data was analyzed by using SPSS (version 25). Statistical significance for the variation in the frequency between groups were determined by Pearson chi-square χ2 test. The *p*-value was calculated at a significance level of (*p* < 0.05).

## Results

Stool samples were examined macroscopically, and the consistency of formed (*n* = 73) and soft to loose (*n* = 115) represented the samples with no abnormal color, or blood/mucus observed. In addition, there were no gross parasitic stages such as segments of tapeworms or adult worms in samples of any investigated worker. There was no statistically significant difference with the physical characteristics of the stool.

Out of 188 participants, 103 (54.8%) and 85(45%) were female and males respectively, with age range between 22–34 years (26.08 ± 2.67).

**Figure 1:**
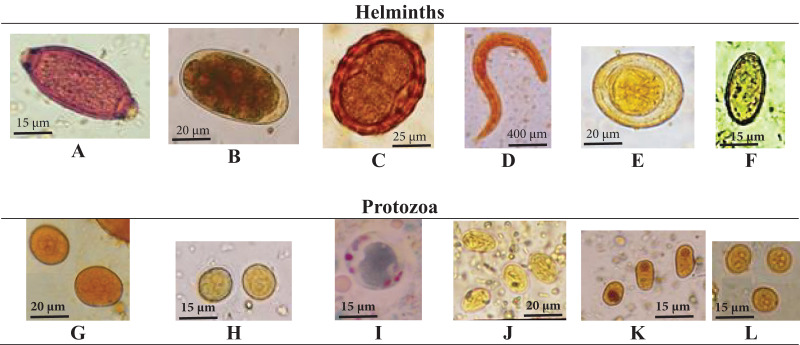
Detected microscopic stages of intestinal parasites. Detected microscopic stages of intestinal parasites including, (A) *T. trichiura* egg; (B) hookworm egg; (C) *A. lumbricoides* egg, (D) *S. stercoralis* rhabditiform larva; (E) *H. nana* egg; (F) *H. heterophyes* egg; (G) *E. coli* cyst; (H) *E. histolytica* cyst; (I) *B. hominis*, (J) *G. lamblia* cyst; (K) *I. buetschlii* cyst; (L) *E. nana* cyst.

Among the analyzed 188 stool specimens by all methods (microscopy, rapid diagnostic test and real-time PCR), 35 samples (18.62%), were infected with intestinal parasites, of which some with multiple infections, including helminths (*T. trichiura*, hookworms (*N. americanus* or *A. duodenale*), *A. lumbricoides*, *S. stercoralis*, *Hymenolepis nana*, *Heterophyes heterophyes*), and protozoa (*E. coli*, *E. histolytica*, *B. hominis, G. lamblia*, *I. buetschlii*, *E. nana*), (see Table 3 and Fig. 1).

**Table 3:** Number of cases infected with single, double, and triple intestinal parasites including helminths and protozoa.

Type of infection	Detected parasites	No. of cases
**Single (26)**	*E. coli* ^1^	7
	*A. lumbricoides* ^1^ ^,^ ^2^	4
	*T. trichiura* ^1^ ^,^ ^2^	3
	*N. americanus* ^1^ ^,^ ^2^	2
	*S. stercoralis* ^1^ ^,^ ^2^	2
	*G. lamblia* ^1^	2
	*A. duodenale* ^1^ ^,^ ^2^	1
	*E. histolytica* ^1^	1
	*H. heterophyes* ^1^	1
	*H. nana* ^1^	1
	*E. nana* ^1^	1
	*B. hominis* ^1^	1
**Double (8)**	*T. trichiura*^1^^,^^2^ *+ N. americanus*^1^^,^^2^	2
	*N. americanus*^1^^,^^2^ *+ B. hominis*^1^	1
	*N. americanus*^1^^,^^2^ *+ E. histolytica*^1^	1
	*E. coli*^1^ *+ E. histolytica*^1^	1
	*E. histolytica*^1^ *+ B. hominis*^1^	1
	*S. stercoralis*^1^^,^^2^ *+ A. duodenale*^1^^,^^2^	1
	*T. trichiura*^1^^,^^2^ *+ S. stercoralis*^2^	1
**Triple (1)**	*T. trichiura*^1^^,^^2^ *+ E. coli*^1^ *+ I. buetschlii*^1^	1

**Notes:**

1 using microscopy.

2 using real-time PCR.

Microscopic examination of stained smears with Kinyoun’s and rapid chromatographic immunoassay test revealed that none of the investigated newly arrived workers was infected with *Cryptosporidium*.

Our investigation of infection types in relation to socio-demographic status, nationality, education level, occupation, symptoms, and other factors are not included in the present study, and will be introduced as a separate study soon.

As shown in Table 4, real-time PCR was able to detect the DNA in (19/188, 10%) samples followed by Ritchie sedimentation technique (18/188, 9.6%) while direct smear was able to detect parasites in only (7/188, 3.7%) of the samples. No statistically significant difference was found among three techniques (*p* > 0.05).

**Table 4:** Summary for the detection of STHs using three techniques (PCR, Ritchie technique, and direct smears).

	Real-time PCR	Ritchie	Direct smears	*p*-value
*Positive*	19 (10.1%)	18 (9.6%)	7 (3.7%)	>0.05
*Negative*	169 (89.9%)	170 (90.4%)	181 (96.3%)	

## Discussion

Many studies on the prevalence of intestinal parasite infections have been done, while a limited number focused on foreign workers in the Kingdom of Saudi Arabia. To the best of our knowledge, this study is the first in Jeddah to investigate the intestinal parasites among newly arrived expatriate labors in Jeddah.

Details of the infection in relation to workers socio-demographic status, nationality, level of education, occupation, symptoms, and other factors are not included here and will be presented soon in a separate study.

In the present study, microscopic examination of direct smear preparations was carried out, followed by the formal ether sedimentation method. This concentration technique enhances the chance of recovery for the diagnostic stages of parasites, mainly in light infections. In addition, real-time PCR was used as a highly sensitive and rapid utility in the detection and identification of STHs (O’Connell & Nutman, 2016; Cunningham et al., 2018).

Other supportive methods including permanent stains and a rapid immunochromatographic kit were used. Permanent trichrome is the most common stain used in parasitology for illustration of morphological features of cysts and trophozoites of intestinal amoebas, flagellates and ciliates. As trophozoites quickly perish, and immediate processing of samples is not always feasible, then permanent staining is the choice.

The prevalence of intestinal parasites in this study was 18.6% among the investigated 188 newly arrived workers in Jeddah. Previous studies conducted among foreign workers in Saudi Arabia reported various prevalence values. Studies in Bahrah, Makkah, Al-Madina, Al-Baha and Al-Khobar reported a prevalence of 22.3%, 16%, 15%, 54%, and 31.4%, respectively (Wakid, 2020a; Ahmed et al., 2015; Taha, Soliman & Banjar, 2013; Mohammad & Koshak, 2011; Abahussain, 2005).

Our study revealed that the total infected cases with intestinal worms were 25 (23 cases with nematoda, one with trematoda and one with cestoda). Some of these cases revealed multiple infections, while the most abundant STHs parasites among the workers were *T. trichiura*, *N. americanus*, *S. stercoralis*, and *A. lumbricoides*. Higher rates of STHs infection were detected in soft and loose samples. This is in agreement with the previous two studies in Al-Madinah that showed *T. trichiura*, *N. americanus*, and *A. lumbricoides* accounted for the highest rates of infections (Taha, Soliman & Banjar, 2013; Imam, Abdulbaqi & Fahad, 2015). According to Bahrah study, all types of STHs were isolated from the investigated samples (Wakid, 2020a).

Real-time PCR in this study showed higher ability to detect the STHs in comparison with microscopic techniques but with no significant differences. This finding is consistent with a previous study conducted in the United Arab Emirates (Al-Rifai et al., 2020), which is due to the differences in sensitivity, specificity, and speed of detection (Ricciardi & Ndao, 2015).

*H. heterophyes* was the only isolated trematoda in our study. On the other hand, *H. nana* was the only detected tapeworm, which was detected in the previous study in Bahrah (Wakid, 2020a).

Among the isolated protozoan parasites from twenty samples, we found that the pathogenic organisms (*E. histolytica* and *G. lamblia*) represented 30%, however the main detected parasites were *E. coli*, *E. histolytica*, and *B. hominis*. Furthermore, the studies in Al-Madinah reported that *G. lamblia, E. histolytica* and *E. coli* accounted for the highest protozoan infections (Taha, Soliman & Banjar, 2013; Imam, Abdulbaqi & Fahad, 2015). According to the study in Bahrah, the most prevalent isolated protozoa were *B. hominis, E. nana, E. coli, G. lamblia* and *E. histolytica* (Wakid, 2020a). *B. hominis* was found to be very common in Makkah study representing 79% of the infected cases, and their finding in contrast to ours (Ahmed et al., 2015). In the past, *B. hominis* had been considered part of the intestinal flora, but recent clinical advances reveal that it is a controversial pathogen (Badparva & Kheirandish, 2020).

Bahrami et al. (2019) showed that direct microscopy is unable to distinguish *E. histolytica* from *E. dispar*. In our study we used the rapid diagnostic test that contains monoclonal antibodies specific for *E. histolytica*, which revealed that all detected four cases were *E. histolytica* and not *E. dispar* (Van den Bossche et al., 2015). Similarly, the detection of the two cases with *G. lamblia* was by microscopic methods and the rapid chromatographic immunoassay test.

Different species of non-pathogenic protozoa can infect humans, and don’t cause symptoms. However, their presence in the stool is an important sign that the patient had ingested some fecal matter, and so their identification has a diagnostic value. In addition, these organisms should ‘ring the alarm”, because they have the same fecal-oral route of infection with the pathogenic organisms (Wakid, 2020a). In our study, three non-pathogenic parasites were identified, including *E. coli*, *I. buetschlii* and *E. nana*.

We believe that the role of large-scale screening among newly arrived expatriate workers by accurate clinical examination and suitable laboratory diagnostic techniques have a significant impact on the sensitivity of parasite identification and therefore on management and control programs of intestinal parasites including STHs.

## Conclusion

The infection of newly arrived foreign workers with intestinal parasites could give rise to a risk for the community. In the current study, almost 19% of the newly arrived expatriate labors had infection with 13 intestinal parasites. There is a need for continuous and regular surveys to handle the occurrence of these infections. For improvement of intestinal parasites identification, we recommend a supportive infrastructure for the application of concentration methods and molecular assays.

## Supplemental Information

10.7717/peerj.16820/supp-1 Supplemental Information 1 Raw data. Raw data for the shown results in Figure 1 and Table 5.

10.7717/peerj.16820/supp-2 Supplemental Information 2 STROBE Statement. STROBE statement checklist of 22 items that are related to the Title and abstract, Introduction, Methods, Results, Discussion, and Other information sections of the article.

## Additional Information and Declarations

### Data Availability

The following information was supplied regarding data availability: The raw data, findings related to another study including infection details in relation to workers sociodemographic status, nationality, education level, occupation, symptoms, and other factors, are available in Tables 1–5.

## References

[ref-1] Abahussain N (2005). Prevalence of intestinal parasites among expatriate workers in Al-Khobar, Saudi Arabia. Middle East Journal of Family Medicine.

[ref-2] Abu-Madi MA, Behnke JM, Ismail A (2008). Patterns of infection with intestinal parasites in Qatar among food handlers and housemaids from different geographical regions of origin. Acta Tropica.

[ref-3] Abubakar I, Tillmann T, Banerjee AJL (2015). Global, regional, and national age-sex specific all-cause and cause-specific mortality for 240 causes of death, 1990-2013: a systematic analysis for the global burden of disease study 2013. Lancet.

[ref-4] Ahmed MA, Alam-Eldin YH, Eltaweel NA, Elmorsy S (2015). Intestinal parasites detected during pre-employment stool examination at tertiary health care center in Makkah, Kingdom of Saudi Arabia. Journal of the Egyptian Society of Parasitology.

[ref-5] Al-Rifai RH, Loney T, Sheek-Hussein M, Zoughbor S, Ajab S, Olanda M, Al-Rasbi Z (2020). Prevalence of, and factors associated with intestinal parasites in multinational expatriate workers in Al Ain City, United Arab Emirates: an occupational cross-sectional study. Journal of Immigrant and Minority Health.

[ref-6] Al-Shammari S, Khoja T, El-Khwasky F, Gad A (2001). Intestinal parasitic diseases in Riyadh, Saudi Arabia: prevalence, sociodemographic and environmental associates. Tropical Medicine & International Health.

[ref-7] Aldahhasi WT, Toulah FH, Wakid MH (2020). Evaluation of common microscopic techniques for detection of Blastocystis hominis. Journal of the Egyptian Society of Parasitology.

[ref-8] Alharbi A, Toulah FH, Wakid MH, Azhar E, Farraj S, Mirza AA (2020). Detection of *Giardia lamblia* by microscopic examination, rapid chromatographic immunoassay test, and molecular technique. Cureus.

[ref-9] Alqarni AS, Wakid MH, Gattan HS (2022). Prevalence, type of infections and comparative analysis of detection techniques of intestinal parasites in the province of Belgarn, Saudi Arabia. PeerJ.

[ref-10] Arfaa F (1981). Intestinal parasites among Indochinese refugees and Mexican immigrants resettled in Contra Costa County, California. The Journal of Family Practice.

[ref-11] Badparva E, Kheirandish F (2020). *Blastocystis hominis*: a pathogenic parasite. Archives of Clinical Infectious Diseases.

[ref-12] Bahrami F, Haghighi A, Zamini G, Khademerfan M (2019). Differential detection of *Entamoeba histolytica*, *Entamoeba dispar* and *Entamoeba moshkovskii* in faecal samples using nested multiplex PCR in west of Iran. Epidem Infection.

[ref-13] Bahwaireth EO, Wakid MH (2022). Molecular, microscopic, and immunochromatographic detection of enteroparasitic infections in hemodialysis patients and related risk factors. Foodborne Pathogens and Disease.

[ref-14] Baker RE, Mahmud AS, Miller IF, Rajeev M, Rasambainarivo F, Rice BL, Takahashi S, Tatem AJ, Wagner CE, Wang L-F, Wesolowski A, Metcalf CJE (2022). Infectious disease in an era of global change. Nature Reviews Microbiology.

[ref-15] Blair I, Sharif AA (2012). Population structure and the burden of disease in the United Arab Emirates. Journal of Epidemiology and Global Health.

[ref-16] Cama VA, Mathison BA (2015). Infections by intestinal coccidia and Giardia duodenalis. Clinics in Laboratory Medicine.

[ref-17] CDC (2019). Amebiasis. https://www.cdc.gov/dpdx/amebiasis/index.html.

[ref-18] Cross J, Basaca-Sevilla V (1981). Intestinal parasitic infections in Southeast Asia. The Southeast Asian Journal of Tropical Medicine and Public Health.

[ref-19] Cunningham LJ, Stothard JR, Osei-Atweneboana M, Armoo S, Verweij JJ, Adams ER (2018). Developing a real-time PCR assay based on multiplex high-resolution melt-curve analysis: a pilot study in detection and discrimination of soil-transmitted helminth and schistosome species. Parasitology.

[ref-20] Efstratiou A, Ongerth JE, Karanis P (2017). Waterborne transmission of protozoan parasites: review of worldwide outbreaks—an update 2011–2016. Water Research.

[ref-21] Expatriate Population (2018). Expatriate population in Saudi Arabia. https://www.globalmediainsight.com/blog/saudi-arabia-population-statistics.

[ref-22] Garcia LS, Arrowood M, Kokoskin E, Paltridge GP, Pillai DR, Procop GW, Ryan N, Shimizu RY, Visvesvara G (2017). Laboratory diagnosis of parasites from the gastrointestinal tract. Clinical Microbiology Reviews.

[ref-23] General Authority for Statististics (2021). Saudi Arabia. https://www.stats.gov.sa/en.

[ref-24] Imam NF, Abdulbaqi ZB, Fahad RA (2015). The prevalence of intestinal parasitic infections among foreign workers in Madinah, Kingdom of Saudi Arabia. Saudi Journal of Medicine & Medical Sciences.

[ref-25] Mejia R, Vicuña Y, Broncano N, Sandoval C, Vaca M, Chico M, Cooper PJ, Nutman TB (2013). A novel, multi-parallel, real-time polymerase chain reaction approach for eight gastrointestinal parasites provides improved diagnostic capabilities to resource-limited at-risk populations. American Journal of Tropical Medicine and Hygiene.

[ref-26] Mohammad KA, Koshak EA (2011). A prospective study on parasites among expatriate workers in Al-Baha from 2009-2011, Saudi Arabia. Journal of the Egyptian Society of Parasitology.

[ref-27] Omrani VF, Fallahi S, Rostami A, Siyadatpanah A, Barzgarpour G, Mehravar S, Memari F, Hajialiani F, Joneidi Z (2015). Prevalence of intestinal parasite infections and associated clinical symptoms among patients with end-stage renal disease undergoing hemodialysis. Infection.

[ref-28] O’Connell EM, Nutman TB (2016). Molecular diagnostics for soil-transmitted helminths. American Journal of Tropical Medicine and Hygiene.

[ref-29] QIAamp (2020). QIAampR fast DNA stool mini handbook. https://www.qiagen.com/us/resources/resourcedetail?id=2a3f2c0b-2e8a-49fd-b442-829108ae1a4a&lang=en.

[ref-30] Ricciardi A, Ndao M (2015). Diagnosis of parasitic infections: what’s going on?. Journal of Biomolecular Screening.

[ref-31] Salas SD, Heifetz M, Barrett-Connor M (1990). Intestinal parasites in Central American immigrants in the United States. Archives of Internal Medicine.

[ref-32] Stephenson LS, Latham MC, Ottesen EA (2000). Malnutrition and parasitic helminth infections. Parasitology.

[ref-33] Taha HA, Soliman MI, Banjar SA (2013). Intestinal parasitic infections among expatriate workers in Al-Madina Al-Munawarah, Kingdom of Saudi Arabia. Tropical Biomedicine.

[ref-34] Taniuchi M, Verweij JJ, Noor Z, Sobuz SU, Van Lieshout L, Petri WA, Haque R, Houpt ER (2011). High throughput multiplex PCR and probe-based detection with Luminex beads for seven intestinal parasites. American Journal of Tropical Medicine and Hygiene.

[ref-35] The World Bank (2021). Population growth (annual %)-Saudi Arabia. https://data.worldbank.org/indicator/SP.POP.GROW?locations=SA.

[ref-36] Torgerson PR, Devleesschauwer B, Praet N, Speybroeck N, Willingham AL, Kasuga F (2015). World health organization estimates of the global and regional disease burden of 11 foodborne parasitic diseases, 2010: a data synthesis. PLOS Medicine.

[ref-37] UNICEF (2014). Migration profiles report. https://esa.un.org/miggmgprofiles/indicators/files/saudiarabia.pdf.

[ref-38] United Nation (2021). International migration 2020 highlights. https://www.un.org/development/desa/pd/sites/www.un.org.development.desa.pd/files/undesa_pd_2020_international_migration_highlights.pdf.

[ref-39] Van den Bossche D, Cnops L, Verschueren J, Van Esbroeck M (2015). Comparison of four rapid diagnostic tests, ELISA, microscopy and PCR for the detection of *Giardia lamblia*, *Cryptosporidium* spp. and *Entamoeba histolytica* in feces. Journal of Microbiological Methods.

[ref-40] Varkey P, Jerath AU, Bagniewski S, Lesnick T (2007). Intestinal parasitic infection among new refugees to Minnesota, 1996-2001. Travel Medicine and Infectious Disease.

[ref-41] Wakid MH (2020a). Prevalence of enteroparasites among non-Saudis in Bahrah, Saudi Arabia. Cureus.

[ref-42] Wakid MH (2020b). Human soil-transmitted helminths and lung infections: a guide review for respiratory therapists. Dr. Sulaiman Al Habib Medical Journal.

[ref-43] Wang L-C (1998). Parasitic infections among Southeast Asian labourers in Taiwan: a long-term study. Epidemiology and Infection.

[ref-44] Ward HD (2009). Intestinal protozoal parasites and diarrheal disease in Bangladesh.

[ref-45] World Health Organization (2002). Prevention and control of schistosomiasis and soil-transmitted helminthiasis: report of a WHO expert committee. http://www.who.int/intestinal_worms/resources/who_trs_912/en/.

[ref-46] World Health Organization (2006). Guidelines for drinking water quality. https://www.who.int/water_sanitation_health/gdwqrevision/cryptodraft2.pdf.

[ref-47] World Health Organization (1997). Amoebiasis. https://iris.who.int/bitstream/handle/10665/230092/WER7214_97-99.PDF.

[ref-48] World Health Organization (2019). Bench aids for the diagnosis of intestinal parasites. https://www.who.int/publications/i/item/9789241515344.

[ref-49] World Health Organization (2020). Soil-transmitted helminth infections. https://www.who.int/en/news-room/fact-sheets/detail/soil-transmitted-helminth-infections.

